# GM2-GM3 gangliosides ratio is dependent on GRP94 through down-regulation of GM2-AP cofactor in brain metastasis cells

**DOI:** 10.1038/s41598-019-50761-5

**Published:** 2019-10-02

**Authors:** Carmen Bedia, Miriam Badia, Laia Muixí, Thierry Levade, Romà Tauler, Angels Sierra

**Affiliations:** 1 Laboratory of Molecular and Translational Oncology, Institut d’Investigacions Biomèdiques August Pi i Sunyer-IDIBAPS, Centre de Recerca Biomèdica CELLEX, Barcelona, E-08036 Spain; 2 Biological Clues of the Invasive and Metastatic Phenotype Group, Bellvitge Biomedical Research Institute (IDIBELL), L’Hospitalet de Llobregat, Barcelona, E-08908 Spain; 3 INSERM UMR 1037, Centre de Recherches en Cancérologie de Toulouse (CRCT), 31037, Toulouse, France; 4 Department of Environmental Chemistry, Institute of Environmental Assessment and Water Research (IDAEA-CSIC), Barcelona, Spain; 5 Centre d’Estudis Sanitaris i Socials-CESS, University of Vic - Central University of Catalonia (UVic-UCC), Vic, E-08500 Spain

**Keywords:** Lipidomics, Metastasis, Mass spectrometry

## Abstract

GRP94 is an ATP-dependent chaperone able to regulate pro-oncogenic signaling pathways. Previous studies have shown a critical role of GRP94 in brain metastasis (BrM) pathogenesis and progression. In this work, an untargeted lipidomic analysis revealed that some lipid species were altered in GRP94-deficient cells, specially GM2 and GM3 gangliosides. The catalytic pathway of GM2 is affected by the low enzymatic activity of β-Hexosaminidase (HexA), responsible for the hydrolysis of GM2 to GM3. Moreover, a deficiency of the GM2-activator protein (GM2-AP), the cofactor of HexA, is observed without alteration of gene expression, indicating a post-transcriptional alteration of GM2-AP in the GRP94-ablated cells. One plausible explanation of these observations is that GM2-AP is a client of GRP94, resulting in defective GM2 catabolic processing and lysosomal accumulation of GM2 in GRP94-ablated cells. Overall, given the role of gangliosides in cell surface dynamics and signaling, their imbalance might be linked to modifications of cell behaviour acquired in BrM progression. This work indicates that GM2-AP could be an important factor in ganglioside balance maintenance. These findings highlight the relevance of GM3 and GM2 gangliosides in BrM and reveal GM2-AP as a promising diagnosis and therapeutic target in BrM research.

## Introduction

The endoplasmic reticulum (ER) has emerged as a major site of cellular homeostasis by harbouring a refined network of molecular chaperones, which acts as a quality control mechanism for homeostatic synthesis, folding and glycosylation of nascent proteins, either secreted from the cell or transported to the plasma membrane^1^. When the efficiency of secretory protein folding is threatened, unfolded/misfolded proteins are improperly glycosylated. These proteins are accumulated and the cell goes through ER stress (ERS) which elicits a homeostatic unfolded protein response (UPR). This response produces then signal transduction cascades that attempt to restore cellular functions^2^. UPR has at least three ER proximal transducers acting as primary molecular sensors: PKR-related ER Kinase (PERK), inositol-requiring enzyme-1 (IRE1), and ATF6^3^.

Among ER chaperons, glucose-regulated proteins (GRP) are characterized by increasing their expression under deregulation of ER-homeostasis induced through hypoxia, nutrient limitation, redox glycolytic reactions, stressful conditions in the calcium metabolism and UPR^4,5^. GRP94 (94-kDa glycoprotein, also known as HSP90B1 or grp96), the luminal ER paralog of HSP90, is an ATP-dependent chaperone that often functions as a dimmer and provides a platform for the assembly or oligomerization of loaded protein cargo, as well as for the transport and folding of other proteins^6^. The specialized role of GRP94 in chaperoning a set of clients is addressed to proteins secretion and surface expression, including the Toll-like receptors (TLRs), integrins, IgG, insulin-like growth factors (IGF-I, IGF-II), Wnt co-receptor LRP6 and cell surface TGFβ–anchoring molecule GARP^2,7–10^. GRP94 also exhibits cell surface and secreted forms that facilitate antigen presentation and immune responses^11^.

ER stress responses have been documented in most major types of human cancer, including breast, pancreatic, lung, skin, prostate, brain, and even liquid malignancies^12^. The ability of cancer cells to handle ERS conditions lies in their intrinsic capacity to adapt for cell survival, or alternatively, to initiate an apoptosis or senescence program through ER-associated machinery^1,13^. In this scene, overexpression of GRP94 induces metabolic stress resistance associated with cellular transformation and increased tumorigenicity in a variety of cancer cell lines^14–16^. Many cancers have been reported to overexpress GRP94^17–19^. Indeed, the depletion of GRP94 in a mouse embryonic model showed that this protein, acting as a stress chaperone, is involved in the regulation of pro-oncogenic signaling pathways and cell adhesion^2^.

Recently, we have observed that over-expression of GRP94 at first diagnosis indicates a high risk of brain metastasis (BrM) progression in breast cancer patients. In fact, GRP94 knockdown drastically impaired brain metastasis in mice experimental models, suggesting a critical role of GRP94 in BrM pathogenesis. Our results indicated that GRP94 is able to couple intracellular and extracellular stress pathways to relieve ERS at low glucose conditions, inducing, therefore, a metabolic switch and pro-survival autophagy (Santana-Codina *et al*., submitted). Indeed, the brain is one of the most highly glucose-dependent organs in the body, where astrocytes consume glucose and shunt lactate to neurons^20^. As a consequence, the glucose levels in the brain interstitial space are lower than in blood^21,22^.

Particularly, cancer cells might up-regulate de novo fatty acid synthesis enzymes to supplement the reduced access to exogenous nutrients and/or fatty acids from poor perfusion within the tumor microenvironment^23^. The main role of UPR signaling in lipid metabolism is promoting lipogenesis^24,25^. Recent studies provided evidence of an inverse correlation between levels of oleic and palmitoyl ethanolamide and GRP78 expression in breast carcinoma cells^24^. Also, an increase in long-chain ceramides and upregulation of several enzymes in the biogenesis of ceramides has been reported when GRP94 was abrogated in hepatocytes, either by genetic or pharmacological methods, indicating a GRP94 intervention in lipid metabolism^26^. Therefore, we hypothesized that to support glucose microenvironmental restrictions, BrM cells over-express GRP94 and regulate lipid metabolism. To understand this critical unexplored role of GRP94 in BrM progression, we performed an untargeted lipidomic analysis of GRP94-silenced BrM cells and non-silenced control cells, which revealed, among other changes, an imbalance in gangliosides composition between GRP94 knockdown cells and their non-silenced counterparts. In addition, the catalytic pathway of GM2 resulted affected by the low enzymatic activity of β-Hexosaminidase (HexA), responsible for the hydrolysis of GM2 to GM3, due to a deficiency of the GM2-activator protein (GM2-AP), the cofactor of HexA, suggesting that GM2-AP might be a GRP94 client protein. Overall, we show a new role of GRP94 in regulating these important cell membrane lipid components.

## Material and Methods

### Cell culture and treatments

All cell lines were maintained under standard conditions in DMEM/F12 medium (Invitrogen) supplemented with 10% FBS, 1 mM pyruvate and 2 mM L-glutamine at 37 °C in a humidified 5% CO2 incubator. Cells were routinely tested for mycoplasma contamination by PCR.

BRV5 cells were obtained after 5 *in vitro*/*in vivo* passages injecting cells in the left ventricle as described elsewhere^27^. These cells contain the retroviral vector preGFP-CMV-PLuc performed as described previously^28^. A cell population that uniformly expressed the highest levels of eGFP (BR-eGFP-CMV/Luc) was selected by FACS (MoFlo, Cytomation, Dako) for *in vitro/in vivo* studies.

#### GRP94 protein stable knockdown

5 constructs containing different short hairpin RNA (shRNA) against GRP94 and a non-target shRNA control vector, were obtained commercially from Sigma Aldrich MISSION® shRNA. BrV5CA1-GFP cells were transfected with the 5 shGRP94 constructs or shControl, respectively, using Lipofectamine 2000 (Invitrogen) as a transfection agent. The clonal selection was performed and Western blot and immunofluorescence were used to validate the knockdown of GRP94 (Santana-Codina *et al*., submitted). Cells used in this work are BRV5CA1 CTRL, as non-silenced GRP94 cells used as a reference, and shGRP94-2 and shGRP94-8, used as stable GRP94 knockdown cell lines.

### Untargeted lipidomic analysis of cell extracts

#### Lipid extraction and analysis

BRV5CA1 CTRL, shGRP94-2 and shGRP94-8 cells were harvested by trypsinization, counted, and 1 million cells/sample was centrifuged at 1300 rpm for 3 minutes at 4 °C. Cell pellets were washed twice with cold PBS. Samples were prepared twice to perform two types of lipid extraction: (1) extraction with chloroform/methanol (2:1) that contains intact lipids from the sample; and (2) extraction chloroform/methanol (1:2) with a saponification step that enables the recovery of sphingolipids. The extractions were performed following the same procedure previously described^29^.

Lipid extracts were analysed using Ultra High-Pressure Liquid Chromatography coupled to mass spectrometry detector (UPLC-MS). The instrument consisted of a Waters Acquity (UPLC) system connected to a Waters LCT Premier orthogonal accelerated time of flight mass spectrometer (Waters), operated in both positive and negative electrospray ionization modes. Full scan spectra from 50 to 1500 Da were acquired, and individual spectra were summed to produce data values each of 0.2 s. Mass accuracy and reproducibility were maintained by using an independent reference spray via the LockSpray interference. The analytical column was a 100 × 2.1-mm inner diameter, 1.7 μm C8 Kinetex (Phenomenex). The two mobile phases were phase A: MeOH/H_2_O/HCOOH (74:25:1, v/v) and phase B: MeOH/ HCOOH (99:1, v/v); both contained 5 mM ammonium formate. The flow rate was 0.3 ml/min and the gradient of A/B solvents started at 80:20 and changed to 90:2 in 3 min; from 3 to 6 min remained at 90:10; changed to 99:1 in 6 minutes until min 15; remained 99:1 until minute 18; finally returned to the initial conditions until minute 20. The column was held at 30 °C.

#### Untargeted lipidomic data analysis

Each of the UPLC-MS data files was converted to a CDF format by the Databridge program of MassLynx software. Then, the data sets were imported into a MATLAB computational environment by using the *mzcdfread* and *mzcdf2peaks* functions from the MATLAB Bioinformatics Toolbox. Two differentiated steps were carried out to perform data analysis: first, a MS spectral data compression using the previously described regions of interest (ROI) strategy^30,31^, and second, the application of multivariate curve resolution-alternating least squares (MCR-ALS), a chemometric technique used for the resolution of pure elution profiles and spectra of components from unresolved complex mixtures such the ones resulting from LC-MS analysis^32^. This combined procedure, known as ROIMCR, has been successfully used in different untargeted metabolomics investigations on LC-MS data^29,33,34^. Briefly, ROIs are defined as the most interesting mass traces with significant MS intensity values higher than a predefined signal-to-noise ratio (generally pre-defined as 0.1 to 1% of the maximum MS intensity). These ROI values are also represented by a minimum of consecutive data points in the chromatogram and have a particular *m/z* deviation error. In our case, ROI values have been obtained from the analysis of 18 samples (6 independent samples for the 3 cell lines) simultaneously, using an in-house written MATLAB routine^35^. The threshold value was set to 1% of the MS maximum intensity, the number of occurrences to 5 and the error in m/z values to 0.025. Given the two types of lipid extraction and the two ionization modes used, a total number of 4 matrices containing the information of all the samples were analyzed. As a result, the number of ROI values obtained in these 4 matrices varied from 180 to 255. Once the size of the LC-MS data matrices was reduced, MCR-ALS was applied in order to resolve the pure component (lipid) contributions present in the complex mixtures. Constraints used during ALS optimization were non-negativity for both elution and mass spectra profiles, and spectra equal height. Application of MCR-ALS to the ROI compressed data matrices resulted in the resolution of a number of components (approximately 150 for each matrix), each one represented by a dyad of profiles that described their chromatographic time elution and their mass spectra profiles. From the relative areas of these MCR-ALS resolved elution profiles, it was possible to estimate the relative amounts of the constituents in every analyzed sample. The values of the areas were corrected using the measured areas of the internal standards added to each sample. For each type of ionization mode (positive and negative), the matrices containing the areas of the components resolved in both types of extraction for all the samples were built up. These matrices were first analysed by principal component analysis (PCA)^36^ to check if samples of the three cell lines could be distinguished by their lipidomic profiles. Then, partial least squares-discriminant analysis (PLS-DA) models^37^ were performed considering two classes in each model: BRV5CA1 CTRL vs shGRP94-2 and BRV5CA1 CTRL vs shGRP94-8. Mathew’s Correlation Coefficient (MCC)^38^ was calculated to validate the goodness of each discrimination model. The MCC is a correlation coefficient between the observed and predicted binary classifications; the values move between +1 (perfect prediction) and -1 (total disagreement between prediction and observation). A value of 0 indicates no better classification than random prediction. The Variable Importance in the Projection (VIP) values^39^ obtained from PLS-DA models are indicative of the influence of each variable (lipid) in the separation of classes, being the scores greater than 1, the ones that are important for the discrimination between groups. Only the variables with VIP values greater than 1 were considered as relevant lipids for the discrimination between GRP94-silenced and non-silenced cells, and used to construct the tables and plots. The tentative identification of lipid compounds was carried out using homemade and online databases such as the Human Metabolome Database (HMDB, www.hmdb.ca) and LIPID MAPS (www.lipidmaps.org). Fold changes for each lipid were calculated by dividing the mean area of each of the silenced clones by the mean area of the non-silenced BRV5CA1 CTRL cells.

#### Analytical software

Software used in this work includes MassLynx V 4.1 (Waters) for raw UPLC-TOF data analysis and *cdf* format conversion. For matrix data processing and statistical analyses, home-made routines for ROI^35^ Bioinformatics Toolbox (The Mathworks Inc.), Statistics ToolBox (The Mathworks Inc.), PLS-Toolbox (Eigenvector Research Inc.) and MCR-ALS Toolbox^40^ were used in MATLAB environment (release 2016b, The Mathworks Inc.).

### Determination of β-hexosaminidase activities

Total β-hexosaminidase and β-hexosaminidase A activities were determined in cell lysates using fluorogenic substrates as previously reported^41^. β-hexosaminidase A activity was determined either after heat denaturation using 4-methylumbelliferyl-2-acetamido-2-deoxy-β-D-glucopyranoside or using the specific substrate 4-methylumbelliferyl-6-sulfo-2-acetamido-2-deoxy-β-D- glucopyranoside.

### Protein expression analysis

#### Western-blot

Cells were lysed in a 1% SDS (v/v) extraction buffer containing an anti-protease cocktail (Roche). Protein concentrations were determined using the Bradford assay (MicroBCA, Pierce). After resolution by SDS-PAGE, electrophoresed proteins were transferred to polyvinylidene fluoride (PVDF) membranes that were blocked and probed with the following antibodies: GRP94 (1:200, sc1794, Santa Cruz Biotechnology), HexA (1:100, sc-376777, Santa Cruz Biotechnology), GM2-AP (1:100, sc-514437, Santa Cruz Biotechnology), GAPDH (1:2500, BD Pharmingen) and the corresponding peroxidase-conjugated secondary antibodies at 1:2000. Immunoreactive bands were quantified using a VersaDoc™ (Bio-Rad) Imaging System using the Super Signal west-Pico (Pierce). Molecular weights were established with See Blue Plus2 Pre-stained Standard (Invitrogen).

#### Immunofluorescence (IF)

For immunofluorescence, 1 × 10^5^ cells were seeded in 24 well-plates containing gelatin-coated coverslips and they were fixed after 24 h using 4% paraformaldehyde in PBS 1X for 15 min at 4 °C. Cells were permeabilized using PBS1x-20%FBS containing 0.2% Triton X-100 for 30 min at room temperature. Then, blocking was performed using 5% BSA in PBS 1X for 1 h. The primary antibodies were goat IgG GRP94 (1/800, sc-1794, Santa Cruz Biotechnology), mouse IgM GM2 (1/50, A2582, Tokyo Chemical Industry), mouse IgM GM3 (1/150, A2576, Tokyo Chemical Industry) and rabbit IgG LC3B (1/500, 3868, Cell Signaling). Alexa Fluor® 555 anti-goat IgG, Dylight® 650 anti-mouse IgM and Alexa Fluor® 594 anti-rabbit IgG (Invitrogen) were used as secondary antibodies (1/1000). Hoescht 33342 staining (Invitrogen) was used for nucleus visualization. For colocalization experiments with Lysotracker (Invitrogen), cells were pre-incubated for 90 minutes with a 75 nM solution of Lysotracker in culture medium before starting the immunofluorescence protocol.

### qRT-PCR

Total RNA was extracted from cells using an RNeasy mini kit (Qiagen) according to the manufacturer’s instructions. One microgram of total RNA was used for reverse transcription using a High cDNA Reverse Transcription Kit (Applied Biosystems). Quantitative real-time PCR was performed using a 7900HT Fast Real-Time PCR System (Applied Biosystems) with validated Taqman Gene Expression Assays (Hs00942655_m1, for HEXA and Hs00166197_m1 for GM2A, Applied Biosystems). Data analysis was based on the ΔΔCt method with β-Actin as the housekeeping gene.

### Statistical analysis

The results were expressed as mean ± SD of three independent experiments unless otherwise specified. Welch’s t-test^42^ was used to check whether the differences observed in qRT-PCR and immunofluorescence experiments were statistically significant.

## Results

### The untargeted lipidomic analysis of shGRP94 cells revealed changes in GM2 and GM3 gangliosides

**Figure 1:**
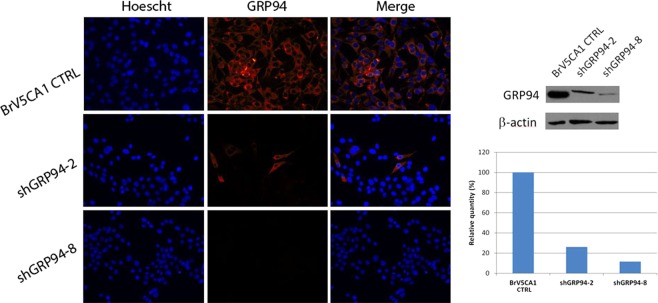
GRP94 expression in BrV5CA1 CTRL, shGRP94-2 and shGRP94-8 cell models used in the present study. (**A**) Immunofluorescence assay of GRP94 expression. (**B**) Western Blot and quantification of GRP94 expression. The bands of the cell clone models used in this work have been cropped from the original gel (available as Fig. S3).

Brain metastatic cells BRV5CA1 CTRL and the stable knockdown shGRP94-2 and shGRP94-8 were used. shGRP94-8 cells showed a more intense GRP94 downregulation than shGRP94-2 cells (Fig. 1A,B). Phenotypically, BRV5CA1 CTRL cells are more resistant than GRP94-ablated cells to glucose deprivation by induction of higher migration abilities and the anti-apoptotic BCL2 (see Supplementary Figs S1, S2, respectively).

**Figure 2:**
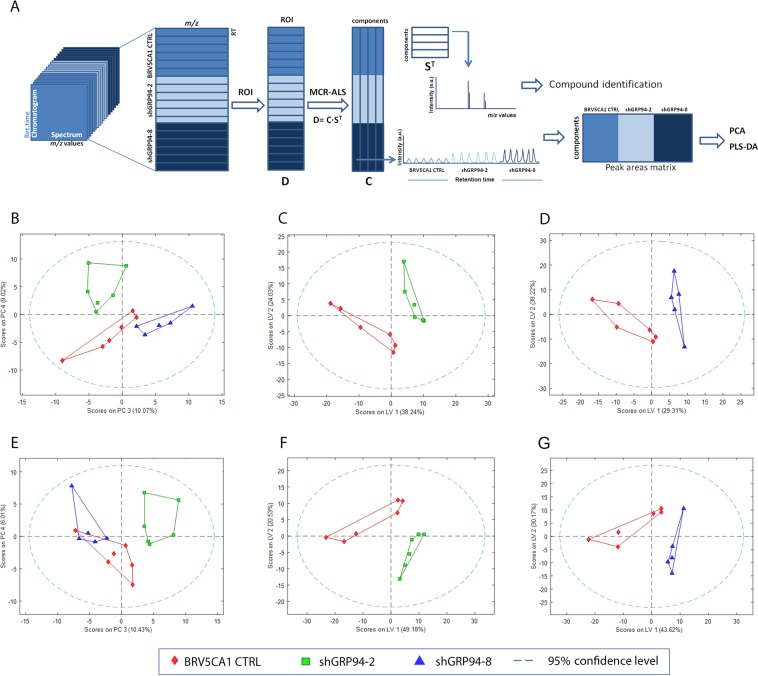
Untargeted lipidomic analysis results, Principal Component Analysis (PCA) and Partial Least-Squares Discriminant Analysis (PLS-DA) of positive and negative ionization lipidomic data. (**A**) Schematic representation of the untargeted lipidomic analysis performed using the ROIMCR procedure, (**B**) PCA of positive ionization analysed data, (**C**) PLS-DA considering BRV5CA1 CTRL and shGRP94-2 classes in positive ionization, (**D**) PLS-DA considering BRV5CA1 CTRL and shGRP94-8 classes in positive ionization, (**E**) PCA of negative ionization analysed data, (**F**) PLS-DA considering BRV5CA1 CTRL and shGRP94-2 classes in negative ionization, (**G**) PLS-DA considering BRV5CA1 CTRL and shGRP94-8 classes in negative ionization data.

Lipid extracts from BRV5CA1 CTRL, shGRP94-2 and shGRP94-8 cell cultures were analysed by UPLC-TOF and the resulting data were further processed by the ROIMCR untargeted analysis strategy, as described in the methodology section and schematically represented in Fig. 2A. Relative concentrations of the resolved lipids resulting from the ROIMCR analysis in both positive and negative ionization modes were analysed by principal component analysis (PCA) (Fig. 2B,E). As shown in Fig. 2B,E, BRV5CA1 CTRL, shGRP94-2 and shGRP94-8 samples could be separated by the variability of PC3 and PC4, indicating that ablation of GRP94 induced changes in the lipid profile of cells. When PLS-DA was applied to see if cells with different GRP94 expression level could be correctly discriminated in the positive mode, shGRP94-2 (Fig. 2C) and shGRP94-8 (Fig. 2D) samples were differentiated from non-silenced ones using two latent variables, explaining 62.2% and 67.5% of the variance, and MCC values of 1 and 0.83, respectively. In the negative ionization mode, the PLS-DA models (shGRP94-2 vs BRV5CA1 CTRL and shGRP94-8 vs BRV5CA1 CTRL, Fig. 2F,G) also gave very good discriminations with MCC values of 1 and 0.83, respectively.

**Figure 3:**
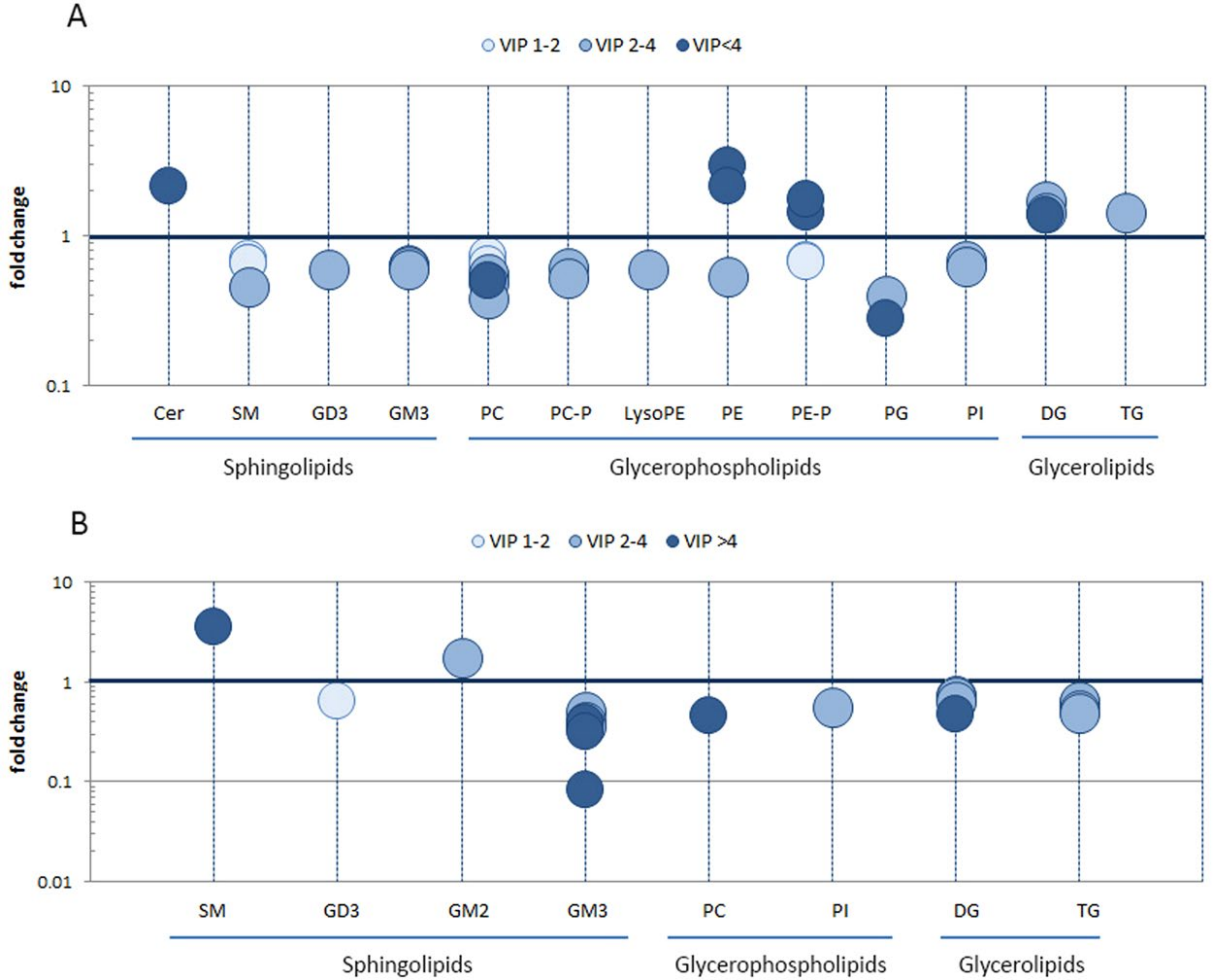
Representative lipid changes in GRP94-silenced clones. Lipid fold changes in (**A**) shGRP94-2 clone and (**B**) shGRP94-clone 8, compared to the control BRV5CA1 CTRL. Only lipids with VIP scores >1 in the corresponding PLS-DA models were used in these plots. Darker circles represent higher influence in the discrimination models built. Cer: ceramide, DG: diacylglycerol, GD3: ganglioside GD3, PC: phosphatidylcholine, PC-P: PC plasmalogen, PE: phosphatidylcholine, PE-P: PE-plasmalogen, PG: phosphatidylglycerol, PI: phosphatidylinositol, SM: sphingomyelin, TG: triacylglycerol.

The variables with VIP values higher than one represented the most influent lipids to discriminate between cell types, and therefore the ones more important to describe the changes in lipid composition due to GRP94 silencing. These lipids are shown in Fig. 3, in which the fold changes in the GRP94 knockdown cells respect to the control are represented for all the selected lipids, ordered by families. Darker circles indicate higher VIP value, indicating more influence in the discrimination. Detailed tables of these lipids, resulting from both comparisons (shGRP94 clone-2 *vs* BRV5CA1 CTRL and shGRP94 clone-8 *vs* BRV5CA1 CTRL) are available in Supplementary Table 1.

As shown in Fig. 3A, similar tendencies can be observed in both GRP94-silenced cell lines with respect to control cells, mainly in glycerophospholipid (GP) and sphingolipid (SL) families. In shGRP94-8 cells, the decrease in GP family is only observed for phosphatidylcholines (PC) and phosphatidylinositols (PI). However, shGRP94-2 cells presented a decrease of several glycerophospholipid species such as PC, PC-plasmalogens, phosphatidylethanolamines (PE), Lyso-PE and PE-plasmalogens, phosphatidylglycerols (PG) and (PI). Most of them are the major constituents of cell membranes, which is in accordance with the fact that the GRP94-silenced cells have reduced proliferation rates. In contrast, in SL family, a 0.6-fold decrease in sphingomyelin (SM) species and a 2-fold increase of Cer (16:0) were observed in the shGRP94-2 clone, which indicated the activation of the sphingomyelinase activity, whereas only an increase of SM (22:0) was noticed in the shGRP94-8 clone. The most interesting result in SLs concerned ganglioside species. In shGRP94-2, three different GM3 species presented decreased levels, with a fold change of 0.6, whereas in shGRP94-8, whose GRP94 expression is even lower than in shGRP94-2, these ganglioside species and four others with shorter chain length were found at lower fold change rates (from 0.1 to 0.5), indicating that this reduction of GM3 levels was related to GRP94 expression. In addition to the decrease of GM3 levels, GM2 species were found increased in shGRP94-8, likely indicating a problem in the metabolic step that linked these two gangliosides. Also, the GD3 ganglioside (d18:0/24:0), was found reduced in the silenced clones. Since GD3 is synthesized from GM3 by the addition of one sialic acid, the reduced levels of GM3 observed might be limiting the production of GD3.

**Figure 4:**
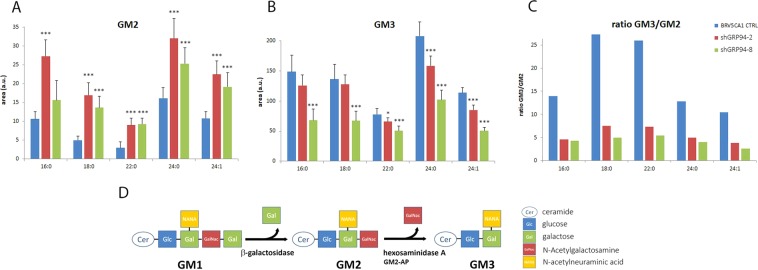
Area quantification of GM2 and GM3 ganglioside species with different acyl length chains in the cellular models. (**A**) GM2 quantification, (**B**) GM3 quantification, (**C**) Representation of the GM3/GM2 species ratio for each cellular model, (**D**) Schematic representation of the GM2 and GM3 biosynthesis. The results are calculated as the mean ± SD of six independent samples. *p < 0.05, ***p < 0.005.

In view of the results, the attention of this study was focused on GM3 and GM2 gangliosides and their relationship to GRP94 expression. Thus, to confirm the observed results, a targeted quantification of GM2 and GM3 species with different acyl chain length was performed on the LC-MS data. The increase of GM2 species was significant independently of the chain length, both for shGRP94-2 and shGRP94-8GM2 (Fig. 4A). In contrast, GM3 species presented a decreasing profile, and it was significant and similar for all the carbon lengths in the clone shGRP94-8, whereas it only was significant for the longest species (22 and 24 carbons) in the case of shGRP94-2. This was traduced to an important decrease of the GM3/GM2 ratio in all ganglioside species with different acyl chain length (Fig. 4C).

### GRP94 regulates lysosomal ganglioside degradation pathway

Gangliosides are glycosphingolipids containing one or more sialic acid (N-acetylneuraminic acid) residues in their carbohydrate moiety^43^. In cells, gangliosides are mainly localized in the outer leaflet of the plasma membrane in lipid-enriched microdomains called lipid rafts where they are involved in cell-cell recognition, adhesion, and signal transduction^43^. In the case of GM2 and GM3, both contain one sialic acid and they are metabolically related by the enzymatic reaction of β-hexosaminidase A (HexA). This heterodimeric enzyme and the cofactor GM2 activator protein (GM2-AP) are responsible for the hydrolysis of the GM2 terminal N-acetyl galactosamine to give GM3^44^ (see Fig. 4D).

**Figure 5:**
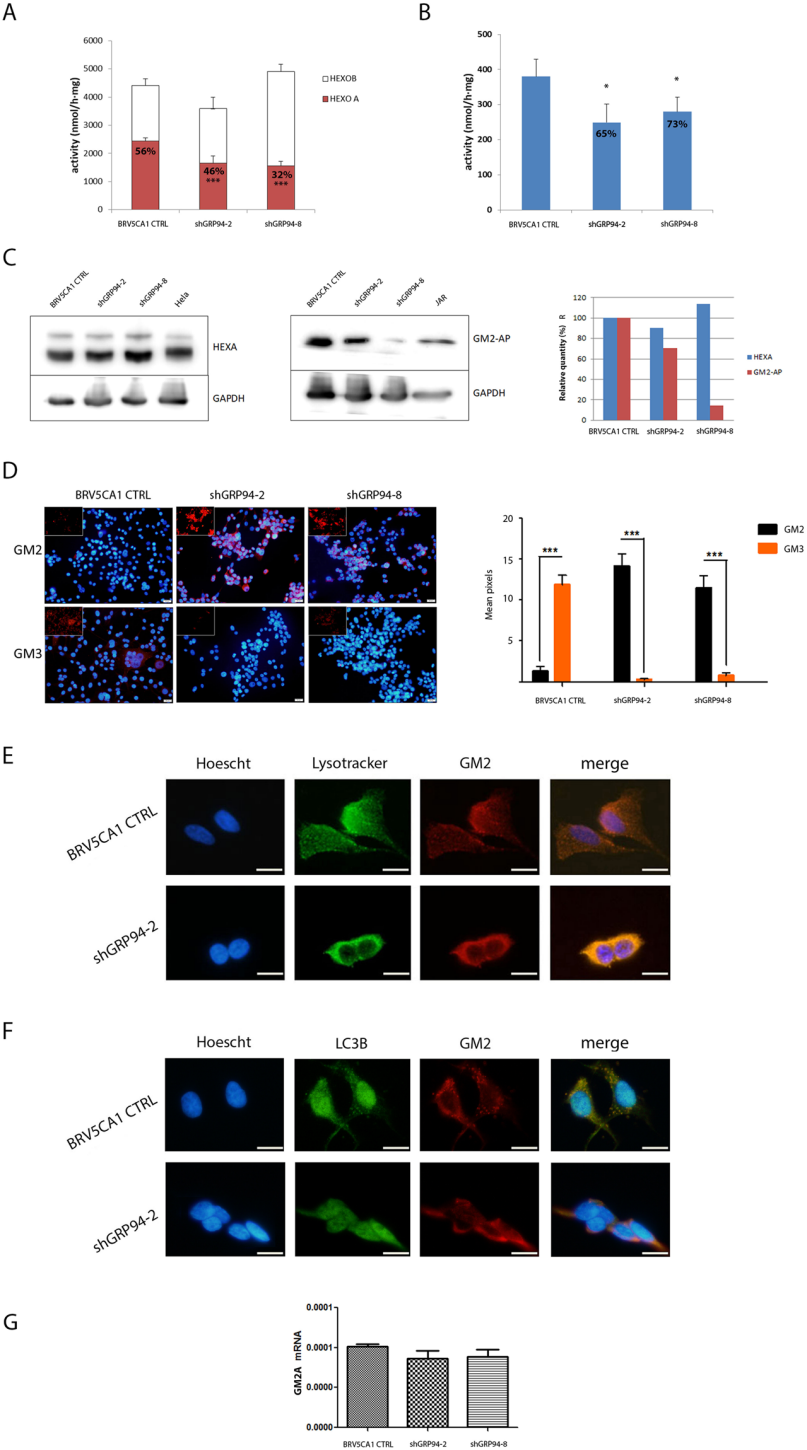
Study of the GM2 hydrolytic process in BRV5CA1 and GRP94-silenced clones. (**A**) HexA, HexB and total activities in the cellular models. HexA activity is calculated by the difference of total activity and HexB activity by thermal denaturation. The percentages of HexA activity respect to the total activity are specified. (**B**) HexA activity measured using the specific substrate for HexA. The percentages of HexA of silenced clones are written down; (**C**) Western Blot for HexA and GM2-AP and relative quantification; Hela cells are used as positive control of HexA, JAR cells are used as a positive control of GM2-AP; the bands of interest have been cropped from the original gel (available as Fig. S4); (**D**) Immunofluorescence assay for GM2 and GM3 and relative quantification. Nuclei were stained in blue and GM2 and GM3 in red, ***p < 0.005; (**E**) Immunofluorescence assay of GM2 and co-localization with Lysotracker, in control and shGRP94-2 cells. Scale bars = 20 µm; (**F**) Immunofluorescence assay of LC3B and GM2, in control and shGRP94-2 cells. Scale bars = 20 µm; (**G**) q-RT-PCR of GM2A gene in the cell clones, ***p < 0.005.

Given the significant decrease of the GM3/GM2 ratio observed in shGRP94-2 and shGRP94-8 cells, the possibility of a blockade of this enzymatic activity was explored. The HexA and the total β-hexosaminidase activities (isoforms A and B) were measured in cells. In the context of the total β-hexosaminidase activity, HexA activity represented 56%, 46% and 32% in BRV5CA1 CTRL, shGRP94-2 and shGRP94-8 cells, respectively (see Fig. 5A). This decrease in the HexA activity was also observed when the specific substrate for HexA (4-methylumbelliferyl-(6-sulfo-2-acetamido-2-deoxy-β-D- glucopyranoside) was used for HexA activity measurement (Fig. 5B), resulting in 65% and 73% of the HexA activity found in BRV5CA1 CTRL for shGRP94-2 and shGRP94-8 cells, respectively. Overall, these results indicated that HexA activity was partially inhibited in GRP94-ablated cells. This HexA activity inhibition invalidated the hypothesis that the accumulation of GM2 in GRP94-deficient cells was due to the overexpression of N-acetylgalactosaminyltransferase (GalNAcT), the enzyme that catalyses the reverse reaction to produce GM2 from GM3. In addition, the gangliosides GD2 and GA2 are also substrates of GalNAcT to produce GD1b and GA1, and any increase in the levels of these gangliosides was found in the GRP94-deficient cell lines.

### GRP94 post-transcriptionally regulates GM2-AP expression

Then, the expression of the alpha subunit of HexA and the GM2-AP cofactor was evaluated in BRV5CA1 CTRL, shGRP94-2 and shGRP94-8 cell lysates. The results evidenced that protein levels of HexA enzyme were stable in all cell lines whereas levels of GM2-AP were critically decreased in shGRP94-2 and almost inexistent in shGRP94-8 (29% and 3% with regard to BRV5CA1 CTRL cells, respectively, Fig. 5C). The decrease of GM2-AP levels was also confirmed by immunofluorescence assay (Fig. S5). These results revealed that the expression variations of GM2 and GM3 and the HEXA activity loss were likely caused by a GM2-AP cofactor deficiency in GRP94-ablated cells.

In order to confirm these results, the presence of GM2 and GM3 in the cell cultures was investigated using immunofluorescence. As shown in Fig. 5D, the concentrations of GM2 clearly increased in shGRP94-2 and shGRP94-8 cells with regard to BRV5CA1 CTRL, whereas almost null expression of GM3 was found in the fluorescence staining. These observations confirmed the previous results that indicated a decreased HexA hydrolytic activity. When the cell distribution of GM2 was explored, a different pattern between BRV5CA1 CTRL and shGRP94-2 cells was observed (Fig. 5E). Whereas GM2 was uniformly distributed within BRV5CA1 CTRL cells, it was more intense and concentrated in shGRP94-2 cells, always colocalizing with the lysosomal distribution. These results suggested that GM2 might be accumulated in the lysosome with consequences on the normal lysosomal functions, such as autophagy. In this context, the presence of LC3B (a marker of autophagosomes) and GM2 was explored in control and shGRP94-2 cells. As shown in Fig. 5F, the colocalization of GM2 and LC3B in control cells indicated the presence of autophagy by fusion of autophagosomes to lysosomes. In contrast, GM2 was increased and accumulated in the lysosomes and LC3B granules were not shown in shGRP94-2 cells.

We checked if a transcriptional activity downstream GRP94 activation was down-regulating GM2-AP expression in GRP94-ablated cells. The results showed no significant change in the expression of GM2-AP cofactor (Fig. 5G), indicating that the reduced levels of GM2-AP in GRP94-ablated cells were due to a post-transcriptional modification.

## Discussion

**Figure 6:**
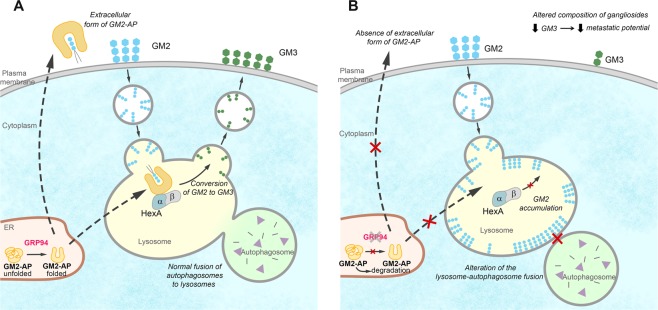
Representation of the hypothesis formulated based on the results of this work. (**A**) Normal expression of GRP94 enables the correct folding of GM2-AP, which, once in the lysosome, catalyses the hydrolysis of the endocyted GM2 to GM3. (**B**) Hypothesis of cell biology in GRP94-silenced cells. The deficiency of GRP94 leads to unfolded/misfolded GM2-AP, which impairs its hydrolytic activity in the lysosome. As a consequence, the endocyted GM2 accumulated in the lysosome and, on one hand, it difficults the autophagosome fusion to the lysosome, and on the other hand, limits the production of GM3, which alters the balance of GM3-GM2 in the plasma membrane, related to the cellular metastatic potential.

Carcinoma cells that survive into the bloodstream and cross the blood-brain–barrier (BBB) suffer a drastic microenvironmental change that has to be countered by metabolic plasticity. In this scene, the glucose-regulated protein GRP94 is critical in BrM success, since GRP94-ablated cells are unable to progress to BrM and, in some cases, after a latency period, they succumb (Santana-Codina *et al*., submitted). Here we report a new function of GRP94 in sphingolipid metabolism that directly affects gangliosides profile in cells. *First*, we show that GM2 ganglioside levels are increased in BRV5CA1 GRP94-ablated cells whereas GM3 decreases, which drastically change the ratio of GM3/GM2 in cells; *second*, levels of β-Hexosaminidase (HexA) activity, responsible for the synthesis of GM3 from GM2, are decreased in GRP94-ablated cells, and *third*, we describe that a post-transcriptional alteration of GM2-activator protein (GM2-AP), the cofactor that interacts with HexA, is responsible for the lower HexA activity observed in BRV5CA1 GRP94-ablated cells. A plausible hypothesis in this context is that GRP94-mediated ER stress response constitutes the central element in the chaperoning process of GM2-AP, promoting cell survival by increasing lysosomal catabolism and autophagy. These functions are impaired in GRP94-ablated cells, in which the down-regulation of GM2-AP might result in the interruption of the GM2 catabolic process in the lysosomes (see Fig. 6A,B).

In the present work, we found higher levels of GM3 in BRV5CA1 CTRL cells with regard to GRP94-ablated cells, which have less metastatic potential (Santana-Codina *et al*., submitted). Thus, GM3 would act as a pro-metastatic lipid in BrM cells, possibly by interacting with neighboring proteins embedded in the plasma membrane that trigger pro-survival and pro-metastatic transduction signals. Although some evidences in literature have related the levels of GM3 to cancer development and progression, the relevance of GM3 in BrM has not been described until now. Our findings agree with Nohara *et al*.^45^ who reported that GM3 was the major ganglioside in MDA-MB-231 cells, a well-known cell model of metastatic breast cancer, and that it was 18-fold increased with respect to the less aggressive and nonmetastatic breast cancer cell line MCF-7. More recently, Kim *et al*. demonstrated that GM3 participates in the epithelial to mesenchymal transition (EMT) process induced by TGF-β1 in lens epithelial cells^46^. Upon EMT activation, GM3 synthase (GM3S) from the GM3 biosynthetic pathway is up-regulated, which in turns increases GM3-serine phosphorylation of TGF-β1 receptors and their interaction, enabling EMT signal transduction and increasing the migration ability of cells. Moreover, GM3S enhanced migration and invasion of murine breast cancer cells, and conversely, GM3-synthase down-regulation suppressed lung metastasis^47^ suggesting that phosphoinositide-3 kinase(PI3K)/Akt pathway is regulated by GM3S.

The present work and these previous studies indicate that an increase of GM3 is associated to cancer progression. However, several lines of study have suggested that GM3 is able to decrease cell motility and growth through modulation of tetraspanins, integrins, and growth factor receptors^48–52^. In addition, GM3 overexpression has been shown to be located on lipid rafts complexed with EGFR and caveolin-1 in human squamous carcinoma cells inhibiting EGFR activation^53^. This controversy has been also reported in breast cancer cells, in which the pre-treatment of MCF-7 cells with exogenous GM3 decreased the stimulatory effect of EGF on cell proliferation^45^. The reported differences in GM3 functions could be explained by the fact that, in some cases, GM3 is added exogenously to different carcinoma cell models. Indeed, tumor cell growth, invasion, and metastasis are complex processes that depend on many factors and not only on ganglioside composition in the membrane. In addition to the chemical composition of lipid rafts, the structural diversity of sphingoid bases and N- acyl chains in the ceramide backbone of gangliosides, and their abundance with respect to other lipids such as phospholipids in the lipid rafts, will determine their chemical interaction with other lipids and neighbor proteins embedded in the membrane. Hence, the lipid and protein profiles should be investigated separately in each cancer cell model in order to assign a specific role of GM3 in cancer progression.

On the other hand, ganglioside catabolism is a stepwise procedure at the surface of luminal intralysosomal vesicle and membrane structures. Hydrolysis of GM2 is facilitated at anionic vesicular surfaces by the cooperation of two proteins, HexA and the membrane lipid-binding protein GM2-AP^54^. The accumulation of unmetabolized substrates in the lysosome, as it occurs in many lysosomal storage disorders (LSDs), results in a variety of pathogenic cascades that can alter some cellular functions such as lipid trafficking and autophagy^55^. Since gangliosides are principal constituents of lipid rafts and play critical roles in membranes physiology by determining their plasticity, it is conceivable that the abnormal accumulation of lipids in the lysosomes leads to changes in lipid rafts that alter the dynamics of lysosomal membranes, specifically their ability to fuse with autophagosomes. Alterations in the autophagic process have been reported in many LSDs^56^, some of them caused by a defective autophagosome-lysosome fusion, such as multiple sulfatase deficiency and Mucopolysaccharidosis type IIA^57^. More experiments are needed to evaluate the functional consequences of the metabolic unbalance of GM2 and GM3 gangliosides on autophagy.

Since GRP94 emerges as a hub towards BrM through the inhibition of apoptosis and activation of pro-survival autophagy in hypoglycemic conditions^1,58^, GRP94 ablated cells have a decreased survival autophagy and down-regulation of Bcl2 (Supplementary Fig. S2), two major factors that favor the adaptation and growth of BrM cells in the brain microenvironment. Therefore, we suggest that GM2 accumulation in the lysosomes might be deleterious for the survival autophagy engagement in BrM cells (see Fig. 6B), in which GM2-AP showed to be a new player in such accumulation.

The crystal structure of mature GM2-AP expressed in *Escherichia coli*^59^, as well as that of lipid complexes of GM2-AP^60,61^, have revealed the protein folding of the enzyme, which contains a hydrophobic pocket able to accommodate the ceramide tail of GM2 ensuring the correct orientation of the tetrasaccharide head-group with respect to the degrading enzyme’s active site. In addition, the structure also contains a single short α helix which has been identified as the major determinant for the interaction with the α subunit of HexA enzyme. One important function of GM2-AP cofactor is to mediate the interaction between the exohydrolase HexA and its membrane-embedded GM2 substrate. GM2-AP is able to recognize GM2 within the membrane plane and to lift it out of the lipid bilayer, thereby presenting it to the water-soluble enzyme for degradation^62^. Thus, the experimental evidence of the present work suggests that GM2-AP might be a client of the chaperone GRP94, since the complex and unique structure of GM2-AP active form might be affected by a defective chaperone folding induced by GRP94 silencing, compromising the hydrolytic activity of HexA.

The inherited deficiency of this cofactor results in the AB-variant form of GM2 gangliosidosis, a rare lipid storage disease that progressively destroys neurons in the brain and spinal cord^63^. In addition to its lysosomal hydrolytic role as a cofactor of HexA, GM2-AP is a secretory protein that can be re-captured by other cells with or without bound lipid, from the extracellular fluid^64^. Due to its unique hydrophobic cavity in its structure, GM2-AP has been shown to bind, solubilize and transport a variety of extracellular lipid molecules, such as glycolipids, gangliosides, and at least one phosphoacylglycerol, between liposomes. Although at pH 7 the lipid transport rate of GM2-AP is reduced only at 50% from its maximum rate achieved at lysosomal pH, this secretory form has been shown to serve as a general intra- and/or inter-cellular lipid transport protein *in vivo*, accelerating their endocytosis and lysosomal degradation^65^. Given the close relationship between ganglioside composition in the membrane and cancer progression and metastasis, the effects of GM2-AP in controlling the amount of these lipids in the cell membrane could be of huge interest in cancer and metastasis research.

In this line, recent publications have proposed GM2-AP as an early lung cancer biomarker^66^, since its expression in both urine and serum was found to be significantly correlated with pathology stage. Moreover, Shin *et al*. established a potential role of GM2-AP in breast cancer progression^67^ due to the elevation of GM2-AP levels found in the secretomes from patients compared to healthy controls. In addition, the siRNA knockdown of GM2-AP decreased migration *in vitro*, whereas the overexpression of GM2-AP induced an increase in cell migration, being these effects more prominent in estrogen receptor-negative cells than in estrogen receptor-positive patients^67^. This is in agreement with the results of the present work and reinforces the hypothesis of a role of GM2-AP in breast cancer metastasis progression, which could be useful to foster the analysis of human samples for stratifying patients who have the risk to develop BrM.

## Conclusions

The results of the present work suggest a new role of GRP94 in maintaining ganglioside catabolism, which is increased in BrM cells. We demonstrated that GRP94-silenced cells presented important changes in the balance of GM2 and GM3 species, which are ultimately caused by a deficiency of the GM2-AP cofactor, necessary for the HexA activity. These observations may have different implications in cell biology (see Fig. 6). Gangliosides strongly control cell surface dynamics and signaling, therefore, it could be assumed that these alterations are linked to modifications of cell behavior acquired by tumor progression. In addition, the lysosomal accumulation of GM2 in GRP94-silenced clones may be related to the autophagy impairment observed in these cells. Altogether, these findings highlight the relevance of GM3 and GM2 gangliosides in BrM and suggest that the cofactor GM2-AP might be a promising diagnosis and therapeutic target in BrM research. Further investigations are needed to elucidate how GM2-AP is able to affect the lipid composition of metastatic and neighboring cells, and also to study the direct role of gangliosides in signal transduction and autophagy in metastasis.

## Supplementary information

Supplementary material

## Data Availability

The data generated or analysed during this study are included in this published article and its Supplementary Information files.
