# Supplementary material for: GM2-GM3 gangliosides ratio is dependent on GRP94 through down-regulation of GM2-AP cofactor in brain metastasis cells
Source: Sci Rep. 2019 Oct 2;9:14241. doi: 10.1038/s41598-019-50761-5 (PMC6775165; doi:10.1038/s41598-019-50761-5)
Supplement: Supplementary file 1 — Supplementary material [file 41598_2019_50761_MOESM1_ESM.pdf]

GM2-GM3 gangliosides ratio is dependent on GRP94 through down-regulation of GM2-AP cofactor in brain metastasis cells

Carmen Bedia[1,4*], Miriam Badia[1], Laia Muixí[2], Thierry Levade[3], Romà Tauler[4] and Angels Sierra[1,5]

SUPPLEMENTARY MATERIAL

-Supplementary figures S1, S2, S3, S4 and S5

-Supplementary tables

Figure S1

Wound healing assay of cell lines used in the present study. A) Representative pictures of the assay. Pictures have been taken after 24, 30 and 48 hours after performing the wound in the confluent cell culture plates. B) Plot representing the percentages of the wound area with respect to the area of the wound at time 0. Results represented the mean ± SD of five independent experiments (n=5). * p<0.05; ** p<0.005

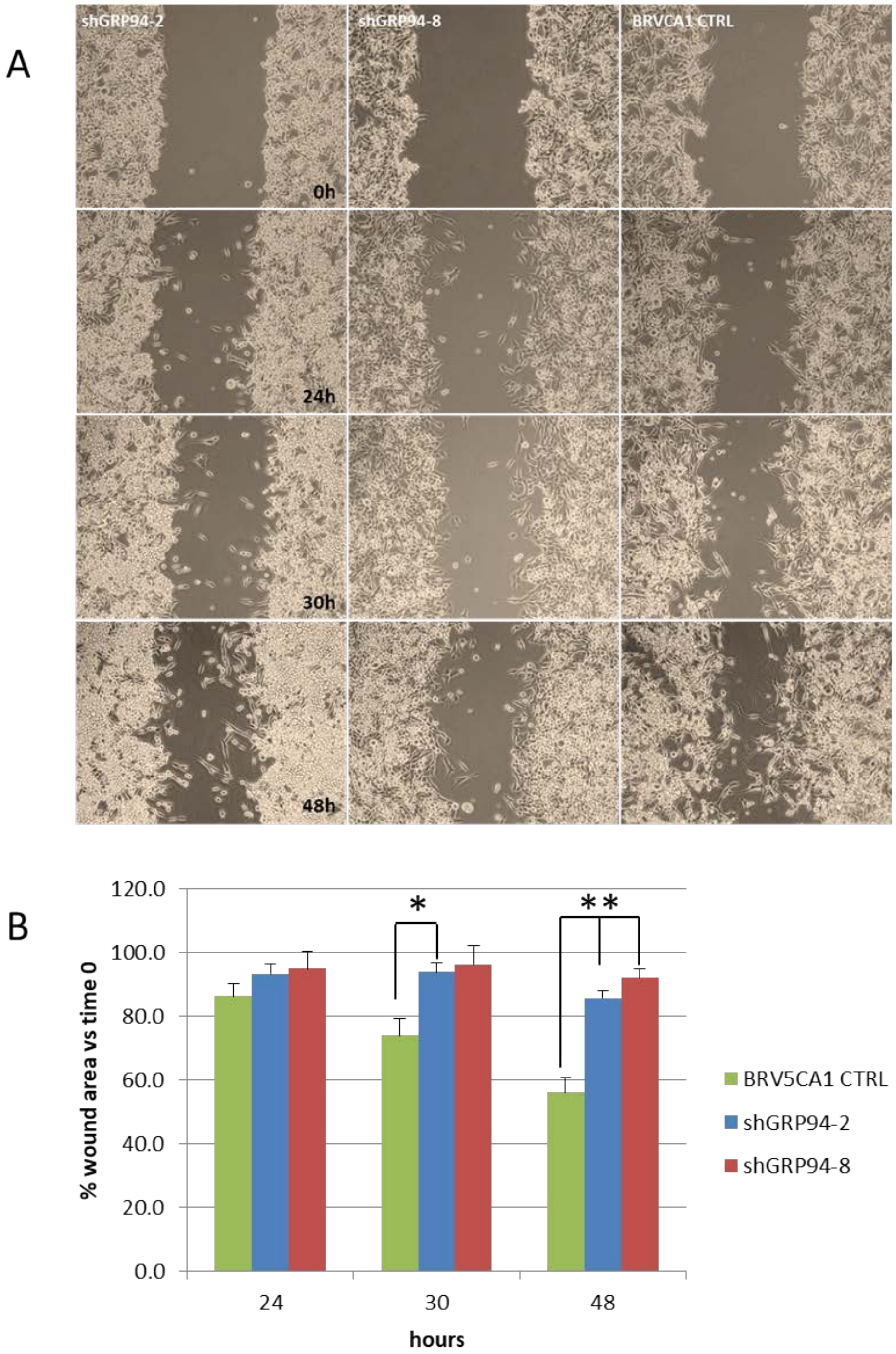

Figure S2

Western Blot of Bcl2 expression in the cell lines used in the present study. The intensity of the Bcl2 bands normalized by the tubulin expression in each lane is represented.

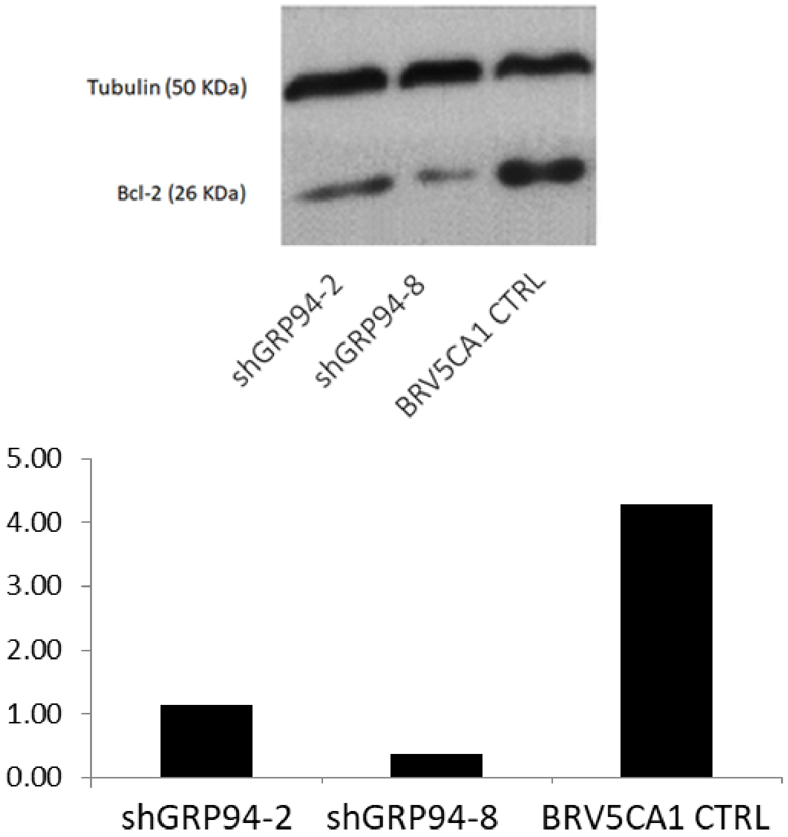

Figure S3

Complete WB gel of GRP94 expression on the different clones isolated after GRP94 silencing. Bands corresponding to the BRVCA1 CTRL, shGRP94-2 and shGRP94-8 cell lines used in the present study are indicated with arrows.

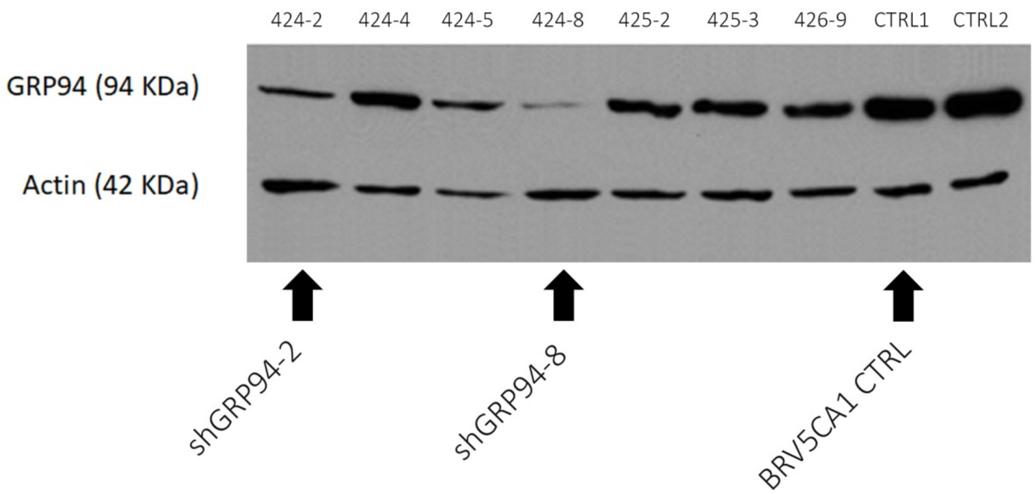

Figure S4

Complete WB gels of GM2-AP and HEXA expression on BRVCA1 CTRL, shGRP94-2 and shGRP94-8 cell lines.

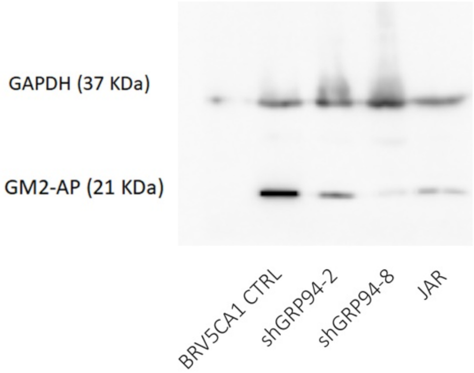

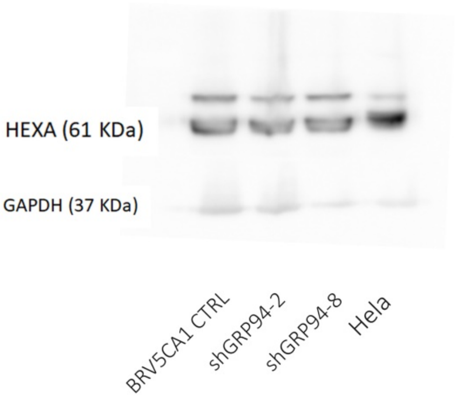

Figure S5

Immunofluorescence assay of GM2-AP expression in BRV5CA1 CTRL and shGRP94-2 cell lines.

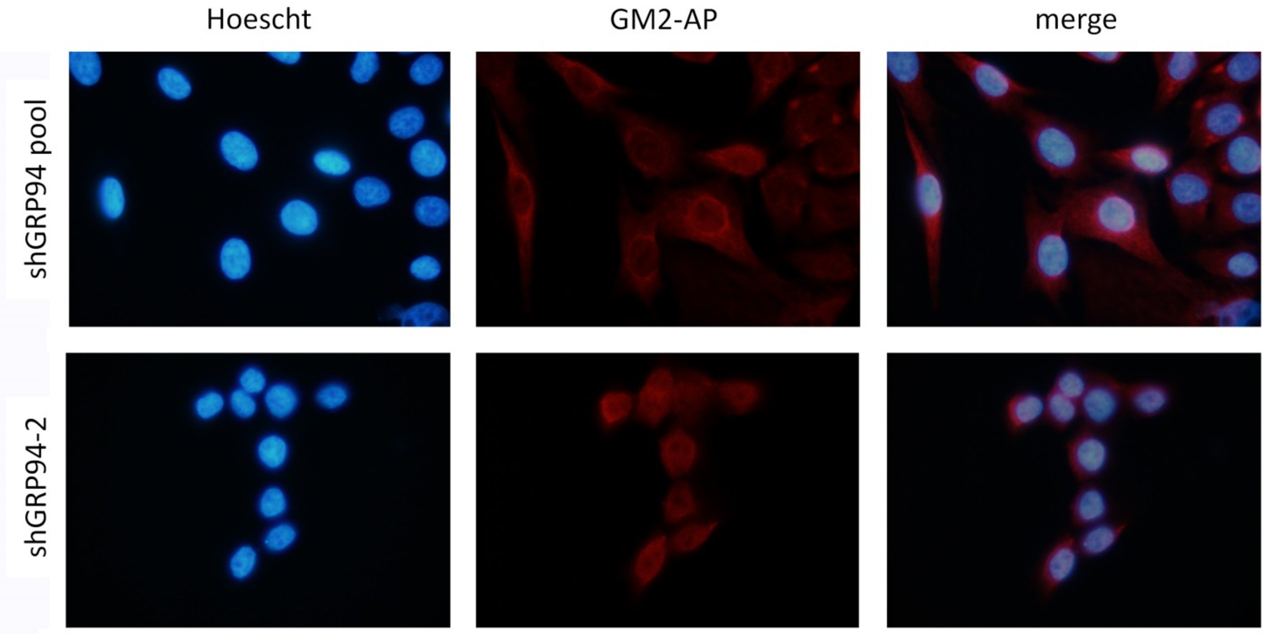

GM2-GM3 gangliosides ratio is dependent on GRP94 through down-regulation of GM2-AP expression: new key players in brain metastasis progression

Carmen Bedia, Miriam Badia, Laia Muixí, Thierry Levade, Romà Tauler and Angels Sierra

SUPPLEMENTARY TABLES

TABLES OF RESULTS OF THE UNTARGETED LIPIDOMIC ANALYSIS PERFORMED USING ROIMCR

Lipid changes in shGRP94 clone 2 with respect to the BRV5CA1 CTRL

Table of results including only the variables with p values < 0.05 and VIP >1

Positive ionization (ESI+)

retention time	m/z	fold change	p value	VIP value	Compound name	Monoisotopic mass	Adduct	Mass adduct	delta error (ppm)	HMDB ID
11.7	555.536	2.2	0.00261	5.0	Ceramide (d18:1/16:0)	537.512	M+NH4	555.546	18	HMDB0004949
5.7	675.544	0.7	0.03281	1.8	SM(d18:1/14:0)	674.536	M+H	675.544	1	HMDB0012097
7.4	690.509	2.9	0.00067	4.8	PE(32:1)	689.500	M+H	690.507	3	HMDB0008924
9.6	813.624	0.5	0.01937	2.3	PE(40:4)	795.578	M+NH4	813.612	15	HMDB0009329
9.7	702.548	1.4	0.02157	4.3	PE(P-34:1)	701.536	M+H	702.543	7	HMDB0009081
9.3	721.560	0.7	0.04691	1.5	PC(30:1)	703.515	M+NH4	721.549	15	HMDB0007902
8.3	767.546	0.4	0.00638	3.0	PC(34:6)	749.500	M+NH4	767.533	16	HMDB0008493
8.3	784.587	0.6	0.02879	1.9	PC(36:3)	783.578	M+H	784.585	3	HMDB0008397
8.1	832.591	0.6	0.03332	1.9	PC(40:7)	831.578	M+H	832.585	7	HMDB0008090
11.8	842.667	0.7	0.03844	1.7	PC(40:2)	841.656	M+H	842.663	4	HMDB0008026
6.6	854.577	0.7	0.02986	1.9	PC(42:10)	853.562	M+H	854.569	9	HMDB0008740
9.7	862.638	0.5	0.02131	2.2	PC(42:6)	861.625	M+H	862.632	7	HMDB0008576
12.8	870.697	0.5	0.00019	4.7	PC(42:2)	869.687	M+H	870.695	3	HMDB0008795
10.9	799.670	0.5	0.01648	2.3	PC(P-38:1)	799.646	M-H2O+NH4	799.668	2	HMDB0011224
10.4	680.515	1.4	0.02935	3.9	DG(40:9)	662.491	M+NH4	680.525	14	HMDB0056308
12.9	600.593	1.7	0.03483	3.2	DG(34:0)	582.559	M+NH4	600.593	1	HMDB0011146
18.8	808.779	1.4	0.01406	3.6	TG(P-48:1)	790.741	M+NH4	808.775	5	HMDB0044646

Negative ionization (ESI-)

retention time	m/z	fold change	p value	VIP value	Compound name	Monoisotopic mass	Adduct	Mass adduct	delta error (ppm)	HMDB ID
6.3	733.5402	0.7	0.03147	2.0	SM(d18:0/14:1(OH))	688.516	M+FA-H	733.514	36	HMDB0013462
7.0	747.5517	0.7	0.04582	1.7	SM(d18:0/16:1)	702.568	M+FA-H	747.566	19	HMDB0013464
10.9	843.6213	0.5	0.01521	3.5	SM(d18:0/22:2(OH))	798.625	M+FA-H	843.623	2	HMDB0013467
9.4	1261.8060	0.6	0.02045	2.7	GM3 (d18:1/24:1)	1262.823	M-H	1261.815	7	HMDB0004848
10.4	1263.8163	0.6	0.02944	2.2	GM3 (d18:1/24:0)	1264.838	M-H	1263.831	12	HMDB0004851
10.7	1265.8272	0.6	0.02024	2.4	GM3 (d18:0/24:0)	1266.854	M-H	1265.847	15	HMDB0011923
9.6	1556.9185	0.6	0.03125	2.1	GD3 (d18:0/24:0)	1557.949	M-H	1556.942	15	HMDB0011866
2.1	500.2758	0.6	0.03964	2.1	LysoPE(20:4)	501.286	M-H	500.278	5	HMDB0011488
7.9	826.5566	0.5	0.01488	2.8	PC(36:4)	781.562	M+FA-H	826.560	5	HMDB0008623
9.6	856.5988	0.6	0.01994	2.6	PC(38:3)	811.609	M+FA-H	856.607	10	HMDB0008594
9.6	798.5975	0.6	0.03269	2.1	PC(P-36:2)	753.604	M+FA-H	798.602	5	HMDB0011334
6.5	732.4779	2.1	0.00072	6.0	PE(32:2)	687.484	M+FA-H	732.482	6	HMDB0008861
8.3	672.4931	1.7	0.00301	6.5	PE(P-32:1)	673.505	M-H	672.497	6	HMDB0011434
7.9	720.5299	0.7	0.04749	1.6	PE(P-32:0)	675.520	M+FA-H	720.519	16	HMDB0001158
10.2	832.6528	0.7	0.04517	1.8	PE(P-40:0)	787.646	M+FA-H	832.644	11	HMDB0011362
10.6	821.5941	0.4	0.01963	2.7	PG(36:1)	776.557	M+FA-H	821.555	48	HMDB0010632
7.6	839.5555	0.3	0.00010	5.9	PG(38:6)	794.510	M+FA-H	839.508	57	HMDB0116629
6.3	809.5141	0.6	0.00727	3.7	PI(32:0)	810.526	M-H	809.519	6	HMDB0009778
6.6	903.5163	0.7	0.01018	2.8	PI(36:4)	858.526	M+FA-H	903.524	9	HMDB0009790
9.7	701.5275	1.4	0.02424	5.4	DG(38:5)	656.538	M+FA-H	701.536	12	HMDB0056212

GM2-GM3 gangliosides ratio is dependent on GRP94 through down-regulation of GM2-AP expression: new key players in brain metastasis progression

Carmen Bedia, Miriam Badia, Laia Muixí, Thierry Levade, Romà Tauler and Angels Sierra

SUPPLEMENTARY TABLES

TABLES OF RESULTS OF THE UNTARGETED LIPIDOMIC ANALYSIS PERFORMED USING ROIMCR

Lipid changes in shGRP94 clone 8 with respect to the BRV5CA1 CTRL

Table of results including only the variables with p values < 0.05 and VIP >1

Positive ionization (ESI+)

retention time	m/z	fold change	p valor	VIP value	Compound name	Monoisotopic mass	Adduct	Mass adduct	delta error (ppm)	HMDB ID
9.9	759.640	3.6	0.0276	5.1	SM(d18:1/20:0)	758.630	M+H	759.638	3	HMDB0012102
12.8	870.697	0.5	0.0022	5.6	PC(42:2)	869.687	M+H	870.695	3	HMDB0008795
10.8	626.575	0.7	0.0390	2.5	DG(35:1)	608.538	M+NH4	626.572	6	HMDB0055991
11.3	638.572	0.6	0.0270	3.2	DG(36:2)	620.538	M+NH4	638.572	0	HMDB0056140
10.9	617.514	0.7	0.0498	2.2	DG(36:4)	616.507	M+H	617.514	0	HMDB0056203
11.3	643.529	0.5	0.0029	4.8	DG(38:5)	642.522	M+H	643.530	0	HMDB0056212
18.2	876.805	0.6	0.0272	2.8	TG(52:2)	858.768	M+NH4	876.802	4	HMDB0045689
17.7	853.730	0.6	0.0267	2.8	TG(52:5)	852.721	M+H	853.728	2	HMDB0048767
19.1	902.821	0.5	0.0174	3.2	TG(54:3)	884.783	M+NH4	902.817	4	HMDB0049122
17.2	881.762	0.6	0.0337	2.6	TG(54:5)	880.752	M+H	881.759	3	HMDB0010460
19.1	907.777	0.5	0.0167	3.2	TG(56:6)	906.768	M+H	907.775	3	HMDB0049456

Negative ionization (ESI-)

retention time	m/z	fold change	p valor	VIP value	Compound name	Monoisotopic mass	Adduct	Mass adduct	delta error (ppm)	HMDB ID
8.0	931.5468	0.6	0.0076	3.4	PI(38:4)	886.5571	M+FA-H	931.5553	9	HMDB0009914
5.3	1151.6986	0.4	0.0045	3.8	GM3 (d18:1/16:0)	1152.7132	M-H	1151.7059	6	HMDB0004844
5.2	1153.7043	0.1	0.0019	4.8	GM3 (d18:0/16:0)	1154.7288	M-H	1153.7215	15	HMDB0011915
6.3	1179.7279	0.4	0.0081	3.4	GM3 (d18:1/18:0)	1180.7445	M-H	1179.7372	8	HMDB0004845
9.1	1235.7906	0.5	0.0179	2.8	GM3 (d18:1/22:0)	1236.8071	M-H	1235.7998	7	HMDB0004847
9.4	1261.8060	0.3	0.0022	4.7	GM3 (d18:1/24:1)	1262.8227	M-H	1261.8154	7	HMDB0004848
10.4	1263.8163	0.4	0.0028	4.4	GM3 (d18:1/24:0)	1264.8384	M-H	1263.8311	12	HMDB0004851
10.7	1265.8272	0.4	0.0023	4.5	GM3 (d18:0/24:0)	1266.8540	M-H	1265.8467	15	HMDB0011923
8.9	1464.8855	1.7	0.0208	2.4	GM2 (d18:1/24:1)	1464.8948	M-H	1465.9021	6	HMDB0004942
9.7	1556.9185	0.7	0.0475	2.0	GD3 (d18:0/24:0)	1557.9494	M-H	1556.9422	15	HMDB0011866
